# Anti-Inflammatory Activity of a Phycocyanin–Protein Complex in THP-1 Cells: Implications for Dermocosmetic Applications

**DOI:** 10.3390/biotech15020045

**Published:** 2026-06-16

**Authors:** Fidel Delgado, Mario Blanco-Vieites, María Álvarez-Gil, Víctor Casado-Bañares, Eduardo Rodríguez

**Affiliations:** 1 Neoalgae Micro Seaweed Products, Calle Carmen Leal Mata, 191, 33211 Gijon, Spain; fdelgado@neoalgae.es (F.D.); malvarez@neoalgae.es (M.Á.-G.); vcasado@neoalgae.es (V.C.-B.); 2 Department of Construction and Manufacturing Engineering, University of Oviedo, Pedro Puig Adam, s/n, 33203 Gijon, Spain; 3 Group of Environmental Studies Applied to Natural and Cultural Heritage, GEMAP (GI-1243), Department of Edafology and Chemical Agronomy, Pharmacy Faculty, Santiago de Compostela University, 15782 Santiago de Compostela, Spain; 4 CRETUS, Santiago de Compostela University, 15782 Santiago de Compostela, Spain

**Keywords:** microalgae, phycocyanin, *Limnospira*, *Spirulina*, anti-inflammatory effects, cosmetics, natural ingredients

## Abstract

Phycocyanin, a phycobiliprotein derived from the cyanobacterium *Limnospira* (*Arthrospira*) *platensis* (commonly known as Spirulina), is recognized for its antioxidant, immunomodulatory, and bioactive properties. This research aims to develop a new cosmetic ingredient based on phycocyanin incorporated into a high-lipid matrix, such as shea butter. A comprehensive characterization of the cytotoxicity and anti-inflammatory activity of this new bioactive phycocyanin–protein complex in human THP-1 monocytic cells was performed. For this purpose, cytocompatibility was evaluated using MTT assays at concentrations ranging from 10 to 0.0006% *v*/*v*. Anti-inflammatory activity was measured under LPS-induced inflammatory stress by measuring IL-6 and IL-8 secretion using ELISA in PMA-differentiated THP-1 cells treated with non-cytotoxic concentrations (0.04, 0.02, and 0.01% *v*/*v*). A crucial finding was the absence of anti-inflammatory activity at 0.01% *v*/*v*, indicating a minimum effective concentration threshold and, consequently, effective doses. The results of this research indicate that the phycocyanin and shea butter ingredients demonstrate strong cytocompatibility at relevant cosmetic doses and significant anti-inflammatory activity, supporting their suitability for formulations targeting skin sensitivity, erythema reduction, and post-inflammatory recovery.

## 1. Introduction

Throughout each historical period, cosmetics have mirrored the prevailing cultural, societal, and beauty standards of their time. In their earliest forms, all cosmetic products were crafted exclusively with simple natural ingredients, primarily due to the limited chemical knowledge available [1]. Thus, plant, mineral, and animal derivatives were the primary sources of cosmetic substances. It was not until the 20th century that synthetic and chemically modified ingredients became widespread in the cosmetics industry. This novelty was mainly driven by the advancement of modern chemistry, which laid the groundwork for the emergence of a globalized and innovative cosmetic sector.

During the 1990s and early 2000s, the use of natural ingredients in cosmetic formulations began to gain popularity [2]. This trend is likely driven by consumer demand and the efforts of cosmetic manufacturers seeking to differentiate themselves in an increasingly competitive market. However, it was during the 21st century that science and technology became deeply integrated into cosmetic development processes, marking a true transformation and the beginning of what is considered nowadays as the modern cosmetic industry [1].

At the moment, clean labeling and sustainability are the most important claims for the cosmetic industry, which are widely found in marine-sourced ingredients [3]. Now, the beauty industry encompasses a commitment to safety, transparency, environmental responsibility and efficacy, reflecting a broader shift towards more conscious consumerism. Algae and marine ingredients offer untapped potential, are environmentally respectful and do not compete with human food resources [2]. One of the most important characteristics of microalgae and marine algae is that they are rich in bioactive compounds (polyphenols, carotenoids and proteins) with multiple applications [4].

Expedited by growing consumer interest in skin and hair care formulations with natural ingredients, algae are expected to strengthen their position in the industry’s spotlight in the coming future. Furthermore, sustainably sourced and packaged variations in algae, kelp and seaweed are increasingly present in new product formulations. Some of the common benefits of algae as a dermatologic cosmetics ingredient, especially in skin care, are its hydrating, hyperpigmentation-reducing, pore-cleansing, fine-line-reducing and anti-inflammatory functions [5]. Regarding the latter, a wide variety of natural materials possessing robust anti-inflammatory behavior have recently emerged as promising candidates for cosmetic applications; these include polysaccharides isolated from the fermentation broth of *Paecilomyces hepialid* [6], extracts from sea buckthorn [7], and *Dictyophora indusiata polysaccharides* [8]. While these diverse natural agents highlight the industry’s continuous search for bioactive molecules to target skin inflammation, microalgae derivatives offer distinct chemical profiles.

Among the wide range of compounds that can be extracted from algae, phycocyanin is a blue phycobiliprotein that is widely found in the blue–green algae from the genus *Limnospira*. Moreover, it is considered one of the most valuable algae extracts by its CAGR (nearly 10%), and its market value is projected to grow to more than $275 million by the year 2030 [9].

Phycocyanin is a supramolecular protein complex that harvests light and delivers it to the reaction centers of prokaryotic cyanobacteria, playing a key role in photosynthesis [10,11]. It is composed of a central core of allophycocyanin surrounded by several rods of phycocyanin. Also, the blue color of phycocyanin is due to a chromophore called phycocyanobilin, which binds proteins through join bonds [12].

Moreover, in the cosmetics industry, the use of phycocyanin is widespread, and its presence in formulas is often labeled as Spirulina extract [13].

Based on Innova Market Insights data, the fastest-growing categories are Face/Body Cosmetics with a 96% CAGR and Suncare with an 89% CAGR (Compound Annual Growth Rate in the last five years). The strong presence of this ingredient in the cosmetic industry and its growing demand are among the pillars that supported the beginning of this study. Moreover, the product category that uses Spirulina extract the most is Suncare products, with a CAGR of 89.88%, with 124 products that use phycocyanin as an additional ingredient. This demonstrates the changes in consumer preferences for the use of more sustainable products and the demand for novel functional ingredients related to Spirulina extracts [9].

The present scientific publication is based on demonstrating the effects of a new cosmetic ingredient developed from a phycobiliprotein obtained from the cyanobacterium *Arthrospira platensis*. The phycobiliprotein employed in this research to produce the new ingredient was phycocyanin [14]. The strong presence of these ingredients and their growing demand were among the pillars to support the beginning of this study. In the present assay, phycocyanin was extracted from *Arthrospira platensis*, a filamentous cyanobacteria with one of the highest recorded protein contents of any whole food. It is also the most widely commercialized microalgae product in the world [15].

During this research, a new cosmetic ingredient was developed whose only cosmetic matrix ingredient is phycocyanin, which has previously been extracted from *Arthrospira platensis* (also known as Spirulina [16]) to demonstrate its anti-inflammatory capabilities through in vivo tests. For this purpose, a human monocytic cell line (THP-1) originally purchased from ATCC (ref. TIB-202™) was used. In parallel to this trial, tests were carried out to demonstrate the safety of the dermocosmetic ingredient with cytotoxicity tests [17]. The safety and effectiveness of a cosmetic ingredient were essential for the successful marketing of the dermo cosmetic product.

## 2. Materials and Methods

### 2.1. Microalgae Strain and Culture Conditions

**Figure 1:**
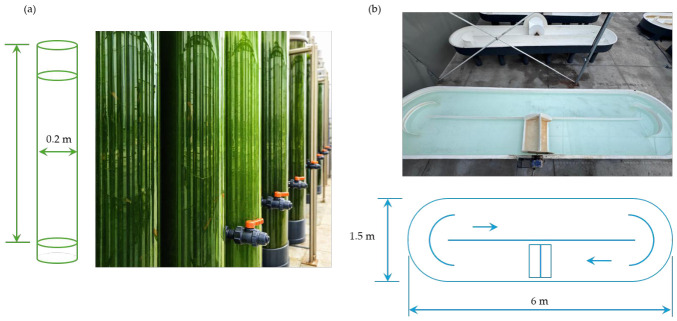
Diagram, dimensions, and images of the closed column-type photobioreactors (**a**) and open-type raceways at Neoalgae (**b**).

The production of this novel cosmetic ingredient necessitated a *Limnospira platensis* species culture. This strain was obtained from the Spanish Algae Bank (BEA) under the accession number BEA 0010B. The cultivation process was maintained under continuous conditions for three months at the Neoalgae Micro Seaweeds Products company, which is situated in Gijon (43.522393139947994; −5.701679544673119). Consequently, the Zarrouk growth medium was utilized as the nutritional formula as it has been tested with good results for cyanobacteria culturing [18,19]. Figure 1 shows the dimensions of the PBRs and raceways employed for cultivation.

### 2.2. Biomass Processing and Phycocyanin Extraction

The cultivation process was meticulously monitored through the implementation of periodic measurements of optical microscopy, spectrophotometric parameters, and dry weight measurements to calculate productivity following the protocols delineated in [20]. THP-1 cells were seeded at a density of 10^6^ cells/mL (10^5^ cells/well in 96-well plates) and allowed to equilibrate for 24 h before LPS stimulation and ingredient exposure. Once the desired cell density was reached, the cultures were harvested by centrifugation using a GMBH model OSE 5-91-037 (GEA Westfalia separator, Oelde, Germany). The biomass obtained was frozen at −40 °C as a pretreatment, followed by lyophilization by freeze–drying to convert the wet biomass into powder.

The Spirulina powder was subsequently utilized as raw material to obtain an aqueous phycocyanin-rich extract. Therefore, it was determined to follow the protocols of extraction delineated in [21] in combination with the drying method established in [14]. Phycocyanin was extracted from the biomass of *Limnospira platensis* (BEA 0010B), previously harvested by centrifugation and frozen at −40 °C. For cell lysis, the frozen biomass was subjected to a freeze–thaw process consisting of repeated cycles of freezing (−18 °C, 24 h) and thawing at room temperature. This physical treatment promotes cell lysis through the formation of ice crystals and membrane rupture, resulting in a phycocyanin-rich fraction. After two freeze–thaw cycles, the lysed biomass was resuspended in distilled water at a biomass-to-solvent ratio of 1:20 (*w*/*v*) and gently mixed for 30 min to facilitate pigment solubilization. The suspension was then subjected to gentle mixing for a period of 30 min with the aim of facilitating pigment. The resulting supernatant, containing the crude phycocyanin extract, was collected and used without further purification, thus avoiding additional costs in the manufacture of the cosmetic ingredient [22]. No chromatographic or precipitation steps were applied, as the objective of the study was to evaluate the biological activity of the crude phycocyanin fraction as an integrator of a cosmetic matrix ingredient.

Phycocyanin is susceptible to thermolability, a property that has thus far impeded its large-scale commercialization, particularly in the field of cosmetics where color is a very important characteristic [23,24]. In order to prevent degradation during the incorporation of the compound into the fatty matrix, it was decided that preservatives should be included in the phycocyanin solution formula. As demonstrated in the safety of certain [10], chemical preservatives were discarded based on their non-safety for human consumption. Consequently, a selection of mono- and disaccharides (glucose, fructose, saccharose, trehalose, lactose, maltose, and sorbitol), inorganic salts (sodium chloride and calcium chloride) and organic acids (citric acid, ascorbic acid, and benzoic acid) was selected [25,26].

In addition to the incorporation of preservatives, a decision was taken to produce an ingredient capable of maintaining the color of the product through the use of shea butter [27,28]. The lipid matrix used in the formulation consisted of refined organic shea butter (certified by COSMOS), supplied by Royal Bio & Natural Ingredients, SL (Murcia, Spain). This material under discussion is obtained by mechanically pressing the seeds of *Butyrospermum parkii*, followed by a refining process, and is intended for cosmetic use (INCI: *Butyrospermum parkii* Butter; CAS 194043-92-0; EINECS 91080-23-8; EC 293-515-7). The following ingredients are contained in the product: *Butyrospermum parkii* butter, crude *Arthrospira platensis* extract (containing 45% C-phycocyanin), trehalose, lecithin, and sodium citrate. This ingredient is a commercial ingredient for the company Neoalgae Micro Seaweeds Products SLU (Gijón, Spain). Based on the proportion of extract and its phycocyanin richness, the final formulation contained 67.3 mg/mL of crude C-phycocyanin complex.

In the ensuing stage of the process, the anti-inflammatory and cytotoxic tests of the compound are conducted, and the compound is incorporated into a neutral cosmetic base; the sole bioactive ingredient is phycocyanin in conjunction with shea butter. Details regarding the specifications and suppliers of all chemical reagents and consumables used in this study are delineated in Table 1.

### 2.3. Conditions of In Vitro Assays

Following the development of the primary functional extract, in vitro functionality was evaluated through in vitro experimental assays at Gaiker facilities (Gaiker Technology Centre, Zamudio, Spain). All laboratory equipment from Gaiker was calibrated following the terms and conditions found under the procedures stated under UNE-EN ISO 9001 [29], UNE-EN ISO/IEC 17025 [30] and GLP (Good Laboratory Practice) [31].

In order to demonstrate the functionality of the developed compound, a cytotoxicity study was conducted to ascertain cell viability after exposure to different concentrations of the compound in a human monocytic cell line (THP-1). Afterwards, an anti-inflammatory activity assay was conducted on the THP-1 cell line.

#### 2.3.1. Cytotoxicity Study

The methodology employed in this study was inspired by the approach documented by [32]; the human monocytic cell line (THP-1) was originally purchased from ATCC (ref. TIB-202™). Cells were maintained in a complete culture medium (RPMI + 25 mM HEPES + 10% FBS + 50 μM β-mercaptoethanol) according to the supplier guidelines and were routinely grown in suspension in tissue culture grade flasks. Subsequent to achieving 80% confluence, the cells were sub-cultured on a new culture onto fresh culture flasks. All the cell cultures were under standard cell culture conditions (37 °C, 5% CO_2_ and 95% humidity). The complete medium contained 100 units of Potassium Penicillin and 100 μg of Streptomycin Sulfate per 1 mL of culture medium. To study the cytotoxicity of the compound a viability assay by Methyl-Thiazolyl Tetrazolium (MTT) was performed in the THP-1 cell line. MTT, a yellow tetrazole, is reduced to purple formazan in living cells, assessing cell metabolic activity by extension, and cell viability.

Cells cultured in 96-well plates were subjected to h15 concentrations of the compound under study for a 24-hour period (37 °C, 5% CO_2_). In parallel, 8 concentrations of a positive control (sodium dodecyl sulfate (SDS)) were also examined in order to validate the assay. After 24 h of incubation with the compound of interest or the positive control, cells were washed with PBS and stained with MTT solution and incubated at 37 °C for 2 h. At the end of this incubation period, the staining medium was removed and 100 µL of DMSO/well was added to solubilize the colored precipitate. The absorbance was read at 570 nm in the spectrophotometer plate reader. The cell viability percentage was calculated relative to the negative control (C−), a condition in which cells were not treated with any compound (as described in Equation (1)):

(1) $$\%\;Cell\;Viability=\left(\frac{AbT}{AbC}\right)\times \;100$$

as AbT absorbance at 570 nm is measured after 24 h of compound exposure and AbC absorbance at 570 nm is measured after 24 h of cells not being exposed to any compound (negative control). For the in vitro experiments, the negative control consisted of the placebo formulation, prepared with the same composition as the cosmetic matrix ingredient but without the phycocyanin-containing extract. The cells consequently were exposed to the carrier system (shea butter and excipients) at the same final concentration that was utilized in the active treatment, ensuring that any observed effect could be attributed exclusively to the phycocyanin complex.

#### 2.3.2. Anti-Inflammatory Activity Assay

The anti-inflammatory activity of the test compound was studied in an in vitro assay in which THP-1 cells were exposed to an inflammatory stimulus (LPS) in the presence or absence of the test compound, as suggested by the findings of [33]. The anti-inflammatory effect of the test compound was measured by quantification of Interleukin-8 (IL-8) and Interleukin-6 (IL-6) secretion levels by ELISA means, as suggested by [34].

Cells were cultivated in 96 well-plates and exposed to 2 ng/mL PMA for monocyte differentiation and attachment to the plate. The next day cells were treated with LPS (50 ng/mL) and different concentrations of the test compound for 24 h. Cells not treated with any product or LPS were used as negative control (C−). Cells treated only with LPS were used as positive control (C+). Following the incubation period, the supernatant of each well was used to quantify the IL-8 and IL-6 production by ELISA (Human IL-8/CXCL8 DuoSet ELISA, R&D; DY208-05 and Human IL-6 DuoSet ELISA, R&D; DY206-05).

## 3. Results and Discussion

### 3.1. Microalgae Cultivation

Microalgae were cultured and maintained throughout the whole experimental assay as a source of biomass for further extraction steps. Laboratory-scale volumes (200 mL to 2 L) were designated as strain cultures and maintained under constant conditions, with the objective of generating a consistent source of inoculum as previously outlined by [35]. Periodic renewals of culture media were done in order to enhance biomass generation in microalgae cultures [36]. Furthermore, the progression of the culture was gauged through periodic measurements of the optical density at absorbance at 680 nm as suggested by [28], until the stationary growth phase was reached (day 10).

The observed results showed that 0.5 L cultures (Figure S1) achieved the stationary growth phase by day 10, which is considered among the optimum range as described by [37]. At this segment of the investigation, absorbance values achieved a maximum of 1.881, which was clearly superior to previously available investigations [38]. On this basis, it was considered that culture conditions satisfied the biological necessities of the selected strain. Thus, further steps included the same culture conditions within the laboratory setting.

At 2 L volumes (Figure S2), cultures demonstrated an average Abs680 value of 0.571, which was replicated by day 4. In comparison with the preceding culture assay, 2 L cultures achieved the stationary phase by day 12, being delayed 2 days more than what was observed in 0.5 L assay. Ref. [39] studied the growth kinetics of *Scenedesmus* and *Chlorella* strains exposed to different substrate turbidities and observed lower maximum absorbance values when compared to the ones presented in this section of our study. However as indicated by the findings of [40], cyanobacteria such as *Arthrospira* typically manifest elevated concentrations of protective pigments, which are mainly visible under absorbance measurements at 680 nm. On this basis, we consider that further work should include a biomass production yield measurement such as dried g/L of culture as suggested by [37]. Moreover, cultures achieved stationary phase by day 12, which was considered the optimum time to proceed with a scale-up, as reported in the findings of [41].

**Figure 2:**
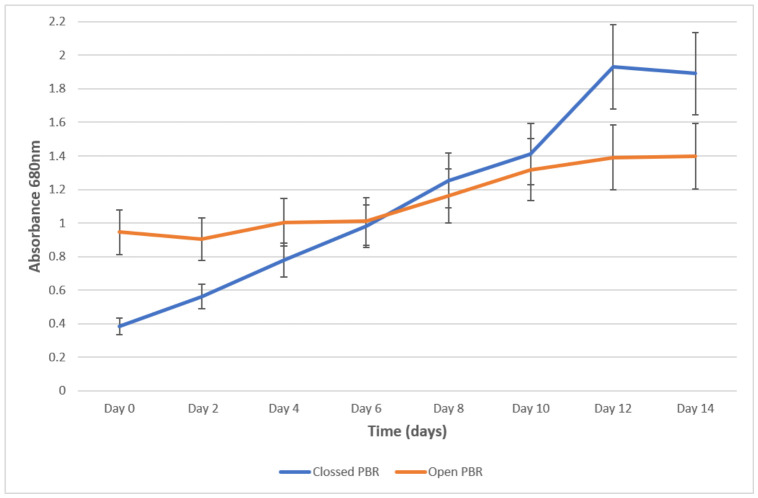
Growth kinetics of pilot-scale culture systems.

Subsequent cultivation steps were conducted in an outdoor greenhouse with uncontrolled conditions of irradiance and temperature. The objective of this experimental phase was to obtain sufficient dry Spirulina biomass to extract a phycocyanin-rich solution from it. This solution was then used to develop a cosmetic ingredient based on phycocyanin as the cosmetic matrix ingredient in the formula. Cultures were maintained within a greenhouse without controlled climate conditions and using column photobioreactors for scaling processes and raceways for large-volume crops. These conditions were stated by previous works as the optimum for the cultivation of cyanobacteria for the strain used during this assay [18]. This step is widely regarded as the most critical one in microalgae cultivation since cultures might collapse as a consequence of the climatic changes, as suggested by [42]. Cultures in 2 L stage were used as inoculum for 100 L vertical photobioreactors (PBRs), which served as future inoculum for open 600 L raceways (open PBR); the growth kinetics of these open PBRs are shown in Figure 2.

The culturing of microalgae and cyanobacteria in both closed and open systems is a subject that is currently being discussed. Closed systems have been shown to offer a robust barrier against external contaminations, while open systems remain sensitive to potential predator microorganisms or competitors. Furthermore, process control is possible in closed systems, while it remains very difficult in open systems, especially in open ponds or raceways. Moreover, reproducibility is challenging when cultures are exposed to uncontrolled ambient parameters, but it is more feasible if closed PBRs are used (Figure 1). Conversely, open-type culture systems exhibit reduced energy requirements and water consumption [43].

Based on the obtained outcomes, it was observed that the selected strain grew with better results in closed PBR, reaching up to 4 times the initial absorbance values by day 12. In contrast, open PBR cultures exhibited a modest augmentation of absorbance, increasing up to 47% of initial values. Nevertheless, both systems proved to be acceptable for *Limnospira* culture, showing significant growth values. Closed PBR growth dynamics demonstrated marked similarity to those similar to what was reported by [44], especially due to the marked exponential growth phase from day 0 to day 8. Opposite to this result, the open PBR assay showed a static growth that is not correlated to the exponential growth phase that is considered as standard in microalgae culturing [45]. Nevertheless, according to the findings of [46], open PBR systems usually show a shortened lag phase that tends to be a linear phase, without entering the standard exponential phase. This can be driven by environmental parameters such as temperature or light variations; however, it should be noted that such variations are not seen in lab-scale culture trials.

Once the raceway achieved the maximum Abs680 observed, prior to the entry of the death-phase of the growth curve, cultures were harvested via centrifugation, obtaining a maximum yield of 2.25 g/L. The findings suggest that the culture protocol and culture conditions were superior to those in other previous works [47,48]. On the same basis, other studies achieved similar production yields [49], who observed a maximum production yield of 2.2 g/L in *A. platensis* open PBR cultures, which is considered comparable to the yield attained in the present study. Nevertheless, these results are still lower than the productivity observed in closed PBR tubular systems, which can reach production yields above 4 g/L [50,51]. On this basis, our findings support that our culture protocol was within the optimum ranges for biomass production. Nevertheless, future work should focus on the potential for enhancing biomass yields and conducting cost–benefit analyses of biomass production.

### 3.2. Limnospira Platensis In Vitro Functional Characterization

The present study was conceived with the objective of evaluating a novel cosmetic ingredient based on phycocyanin extracted from *Limnospira platensis*, with particular attention to both its safety profile and its potential anti-inflammatory activity in THP-1 cells. This approach is especially relevant in the current cosmetic landscape, where there is a strong demand for natural, sustainable, and multifunctional ingredients with demonstrated biological activity. In this particular context, phycocyanin is an attractive candidate because it combines a marine-origin story with bioactive properties that fit well within clean-label and efficacy-driven cosmetic development. It is evident from an examination of the extant literature that previous works have developed phycocyanin emulsions made with mixtures of oil and water and nanoparticles, which also increase stability. Nevertheless, based on the cosmetic target of the development, it was decided not to use any type of nanoparticles and instead a natural compound such as shea butter would be utilized [27,28].

**Figure 3:**
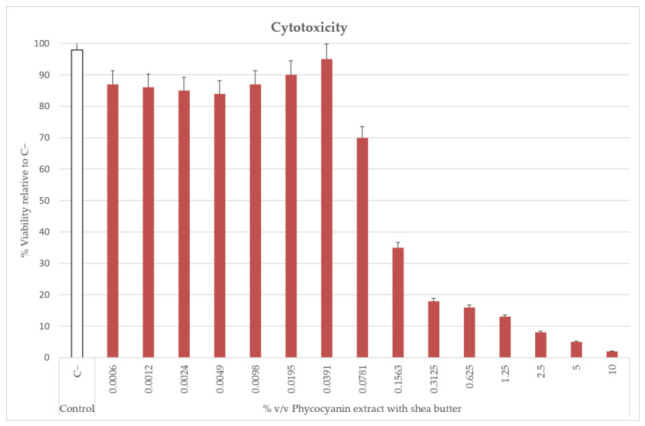
Percentage of cell viability after 24 h of exposure to different concentrations of the test product measured by MTT assay in THP-1 cell line. Bars represent the average of six technical replicates, and error bars correspond to the standard deviation. Negative control (C−): cells not treated with any compound.

The cytotoxicity of the phycocyanin–protein complex under study was evaluated in an in vitro viability assay, MTT assay, in the THP-1 cell line. Fifteen concentrations (10, 5, 2.5, 1.25, 0.625, 0.3125, 0.1563, 0.0781, 0.0391, 0.0195, 0.0098, 0.0049, 0.0024, 0.0012 and 0.0006% *v*/*v*) were tested. The results are represented in Figure 3. The phycocyanin–protein complex shows high cytotoxicity at the upper concentrations tested (10–0.0781%). Subtoxic concentrations (more than 80% cell viability) are reached at 0.0391% and below (0.0391–0.0006%).

In the cytotoxicity assay (MTT test), the compound demonstrated significant cellular toxicity at the higher concentrations evaluated (from 10% to 0.0781% *v*/*v*), resulting in a substantial reduction in cell viability. Concentrations of 0.0391% *v*/*v* and below maintained cell viability above 80%, defining this range (0.0391–0.0006%) as subtoxic concentrations for subsequent biological assays. A distinct safety threshold was apparent at 0.0391% *v*/*v* and below, where cells persisted above 80%.

**Figure 4:**
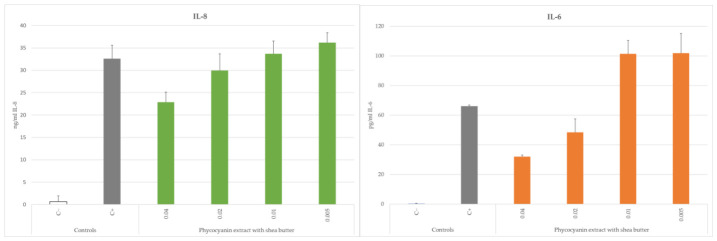
Cytokine (IL-8 and IL-6) secretion after 24 h of exposure to LPS and different concentrations of the test compound in the THP-1 cell line. Bars represent the average of three technical replicates and error bars correspond to the standard deviation. Negative control (C−): cells not exposed to any compound or LPS. Positive control (C+): cells exposed to LPS.

The anti-inflammatory activity of the phycocyanin–protein complex under study was evaluated in an in vitro assay in theTHP-1 cell line. In light of the findings from the cytotoxicity assay results, three concentrations were tested: 0.04, 0.02 and 0.01% *v*/*v*. The results are represented in Figure 4. The compound demonstrated anti-inflammatory activity at the two upper concentrations tested (0.04 and 0.02%). The anti-inflammatory activity displays a dose-dependent pattern, decreasing with the compound concentration. The developed cosmetic product demonstrates a loss of activity at the lower concentration that was tested (0.01%).

In the anti-inflammatory activity assay, based on the results of the cytotoxicity assay, three safe concentrations were selected: 0.04%, 0.02%, and 0.01% *v*/*v* [52]. The compound demonstrated a substantial anti-inflammatory effect at the two highest concentrations (0.04% and 0.02%), evidenced by the reduction in pro-inflammatory cytokines (IL-6 and IL-8) secretion induced by LPS stimulation. These response levels are consistent with those observed in other similar natural products [53]. This effect followed a dose-dependent pattern, gradually decreasing with lower concentrations and completely disappearing at 0.01% *v*/*v*.

The findings indicate that the phycocyanin–protein complex exhibits significant anti-inflammatory activity within a non-cytotoxic concentration range. This supports the hypothesis that the complex has potential application in therapeutic or cosmetic contexts where modulation of inflammatory responses is desired without compromising cell viability [54].

The anti-inflammatory assay further supports the interest in this ingredient. At 0.04% and 0.02% *v*/*v*, the extract reduced the secretion of IL-6 and IL-8 after LPS stimulation, indicating a measurable anti-inflammatory effect in a relevant immune-cell mode. This data obtained agrees with the data from [55], since natural products have gained much importance in controlling inflammatory processes.

It is important to note that this effect was lost at 0.01% *v*/*v*, which suggests that the activity follows a dose-dependent pattern and requires a minimum effective concentration to be biologically relevant. This data agrees with the research carried out on botanical extracts of *Thymus satureioides* leaves wherein it was observed that the compounds in question become inactive at low concentrations [56]. This is a key point for cosmetic formulation, because it indicates that extremely low concentrations may be safe yet inadequate in terms of delivering the anticipated functional benefit.

In the context of cosmetic applications, such a profile is favorable because anti-inflammatory actives are often most valuable when they provide efficacy at low to moderate inclusion levels. Consequently, they can be incorporated into leave-on products intended for sensitive, reactive, or redness-prone skin. Extracts such as tea leaf extract were evaluated by [57] with a control greater than 0.05 s and no activity was found like that of the phycocyanin extract with shea butter.

From a formulation perspective, the results suggest that the final concentration in the cosmetic product should be selected with caution, taking into account both the in vitro effective dose and the extent of skin delivery. Indeed, a number of studies have shown that encapsulating herbal substances like indica in microemulsions can reduce skin irritation caused by the ingredients themselves and thus improve their incorporation into the final formula [58].

However, it was observed that not all of the incorporated active ingredients reached the viable epidermis or dermis. Consequently, the concentration used in the final product was typically chosen to be higher than the concentration that exerted activity in a cell-based assay. In a number of natural cosmetic ingredients, such as calendula extracts, results demonstrated dose-dependent inhibition of nitric oxide (NO) production (up to 50%) with a favorable safety profile, which has confirmed the anti-inflammatory activity of calendula flower extract [59].

In the domain of practical dermocosmetic development, this frequently commences from the biologically active range and makes upward adjustments to compensate for formulation losses, skin barrier limitations, and incomplete penetration. This approach is undertaken while maintaining a safe margin. [60].

From a cosmetic perspective the potential applications of this ingredient could be positioned for applications such as soothing, redness-reducing, post-exposure recovery, sensitive-skin care, and anti-pollution or anti-irritation products. The provenance of the substance from *Limnospira platensis* [61] serves to reinforce its appeal in formulations marketed under natural, sustainable, or marine-based claims, which are increasingly important in the current market.

Moreover, the observation of the cosmetic matrix ingredient at relatively low concentrations may be advantageous from formulation and cost perspectives, as it allows for compatibility with other functional ingredients without requiring a high loading of the extract.

The results of the study demonstrate that the phycocyanin-based extract exhibits dermocosmetic potential, particularly in terms of its anti-inflammatory properties. Future work should ideally include skin penetration, stability, irritation, and final-product performance testing to confirm whether the proposed concentration range’s efficacy within a real cosmetic matrix.

## 4. Conclusions

The present study demonstrates that a phycocyanin-containing ingredient obtained from *Limnospira platensis* and incorporated into a shea butter matrix constitutes a technically robust system for stabilising the crude phycocyanin–protein complex and enabling its application in lipid-based topical formulations. It is evident that the matrix effectively enhanced pigment stability and preserved its chromophoric integrity throughout the process of handling and testing.

Following a comprehensive study and rigorous determination of the cytotoxicity assessment, it was demonstrated that the ingredient developed in this study remains non-cytotoxic at concentrations ≤0.0391% *v*/*v*, thereby establishing a clear safety threshold that coincides with previous findings on the stability and biocompatibility of phycocyanin [14,23]. The data presented herein lend support to the hypothesis that the substance is suitable for incorporation into cosmetic products intended for regular topical use, where cell integrity is of paramount importance for consumer safety [17].

Secondly, and a key finding of this research, it was demonstrated that within the non-cytotoxic range of the ingredient, concentrations of 0.04% and 0.02% *v*/*v* significantly reduced LPS-induced IL-6 and IL-8 secretion in THP-1 cells.

The observed absence of activity at 0.01% *v*/*v* indicates the presence of a minimum effective concentration, a pivotal factor in guiding dosage decisions during formulation development.

In consideration of the findings from the present study, the novel phycocyanin and shea butter extract is identified as a promising ingredient for calming and anti-redness applications, for sensitive skin, and for post-exposure care. The product’s natural and sustainable provenance, coupled with its comprehensive natural index, corresponds seamlessly with contemporary demands within the cosmetics market. Consumers are progressively seeking natural bioactive ingredients that have been scientifically validated for their efficacy [1]. The results obtained demonstrate the technological relevance and innovation of this lipid matrix developed in this research. This development functions not solely as a stabilizing agent but also as a way to incorporate phycocyanin into fatty bases for use in cosmetic formulations.

Consequently, the platform developed in this study offers a versatile and scalable approach for incorporating hydrophilic-sensitive active ingredients into modern cosmetic formulations, ensuring the preservation of both efficacy and sensory quality.

## Figures and Tables

**Table 1:** Chemical reagents and consumables used during experimental assay and providers.

Reagents and Consumables	Provider
RPMI-1640	SIGMA (St. Louis, MO, USA); R0883
Fetal bovine serum (FBS)	GIBCO (Glasgow, UK); 10106-169
β-mercaptoethanol	SIGMA; M3148
Penicillin/streptomycin (P/S)	GIBCO; 15140-122
Phorbol 12-myristate 13-acetate (PMA)	SIGMA, P1785
Trypsin-EDTA 10×	SIGMA; T4174
PBS 10×	ROCHE (Basel, Switzerland); 11666789001
Hanks’ Balanced Salts	SIGMA; H1387
Trypan Blue solution	GIBCO; 15250-061
Sodium dodecyl sulfate (SDS)	SIGMA; L6026
MTT. CAS No 298-93-1	SIGMA; M2128
Dimethyl sulfoxide (DMSO)	PANREAC ITW REAGENTS (Barcelona, Spain); 161954.1612
E. coli lipopolysaccharide (LPS)	SIGMA; L6529
Human IL-8/CXCL8 DuoSet ELISA	R&D (Minneapolis, MN, USA); DY208-05
Human IL-6 DuoSet ELISA	R&D; DY206-05
DuoSet ELISA Ancillary Reagent Kit	R&D; DY008B
HEPES buffer	SIGMA; H3375

## Data Availability

The original contributions presented in this study are included in the article. Further inquiries can be directed to the corresponding author.

## Supplementary Materials

The following supporting information can be downloaded at: https://www.mdpi.com/article/10.3390/biotech15020045/s1, Figure S1: Growth kinetics of lab-scale cultures in 0.5 L; Figure S2: Growth kinetics of lab-scale cultures in 2 L.
