# Supplementary material for: Anti-Inflammatory Activity of a Phycocyanin–Protein Complex in THP-1 Cells: Implications for Dermocosmetic Applications
Source: BioTech (Basel). 2026 Jun 16;15(2):45. doi: 10.3390/biotech15020045 (PMC13296759; doi:10.3390/biotech15020045)
Supplement: Supplementary file 1 [file biotech-15-00045-s001.zip › biotech-4348919-supplementary.pdf]

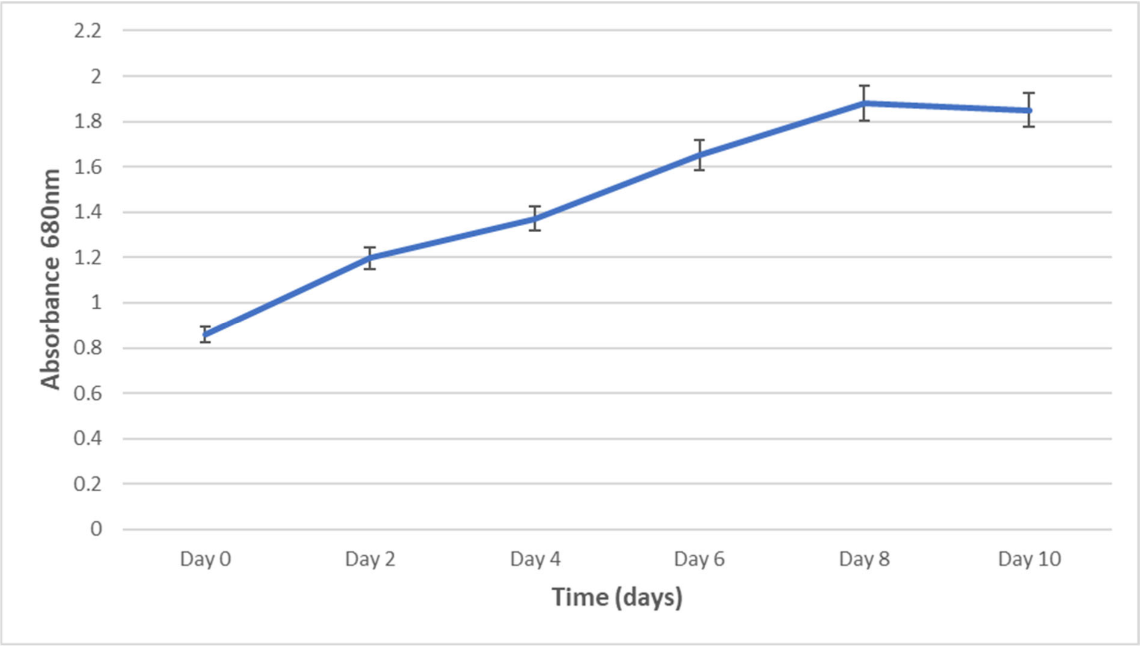

Figure S1. Growth kinetics of lab-scale cultures in 0.5 L

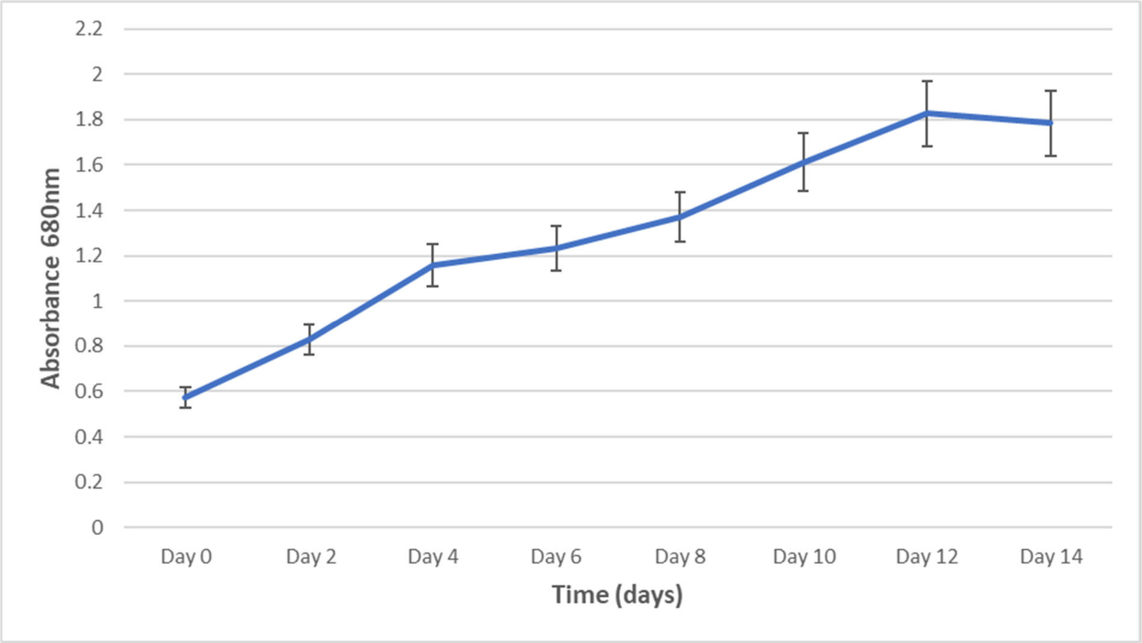

Figure S2. Growth kinetics of lab-scale cultures in 2 L.
